# Hyper-Thyro Vision: An Integrated Framework for Hyperthyroidism Diagnostic Facial Image Analysis Based on Deep Learning

**DOI:** 10.3390/biomimetics11030210

**Published:** 2026-03-15

**Authors:** Poonyisa Thepmangkorn, Suchada Sitjongsataporn

**Affiliations:** 1 The Electrical Engineering Graduate Program, Faculty of Engineering and Technology, Mahanakorn University of Technology, Nongchok, Bangkok 10530, Thailand; 6719720001@mut.ac.th; 2 Department of Electronic Engineering, School of Electrical and Electronic Engineering (SEE), Mahanakorn University of Technology, Nongchok, Bangkok 10530, Thailand

**Keywords:** artificial intelligence (AI), deep learning, hyperthyroidism, exophthalmos, goiter, multi-modal framework, feature fusion, facial images

## Abstract

This paper presents an integrated multi-modal framework for detecting hyperthyroidism-associated abnormalities, namely exophthalmos and thyroid-related neck swelling, through the joint analysis of frontal facial and neck images using a deep learning-based approach. The objective of this research is to develop an integrated AI framework that improves hyperthyroid-related abnormality detection by simultaneously analyzing facial images of both the eye and neck based on pattern clinical knowledge. The multi-modal framework mimics a biological visual mechanism by using a dual-pathway architecture that concurrently processes foveal-like details of the eyes and neck. It integrates these high-resolution visual embeddings with quantitative morphological measurements to simulate a clinician’s ability to fuse observation with physical assessment. The proposed system employs a multi-faceted decision-making process derived from three distinct data components: two from frontal face analysis and one from neck region analysis. Specifically, eye regions extracted from facial images are preprocessed using the YOLOv11s model. The proposed system leverages a dual-pathway processing architecture to extract comprehensive diagnostic features. For the eye dataset, the framework utilizes a face mesh-based eye landmark (FMEL) to extract both eye regions and perform eyes unfold processing. These regions are subsequently analyzed by the proposed sclera map unwrapping engine (SMUE) to derive quantitative sclera metrics from both the left and right eyes. To optimize classification, a dual-branch architecture is employed by integrating CNN visual embeddings with SMUE-derived statistical features through a feature fusion layer. Simultaneously, the neck processing path executes the neck region of interest (ROI) prediction {upper, lower} to segment critical regions for goiter assessment via the proposed neck μ−σ ensemble thresholding (NSET) algorithm. The experimental results demonstrate that the proposed algorithm for eye analysis achieved a mean average precision (mAP50) of 96.4%, with a specific mAP50 of 98.6% for the hyperthyroid class. Regarding quantitative scleral measurement, the SMUE process revealed distinct morphological differences, with the experimental data group exhibiting consistently higher pixel distances across the reference points compared with the normal group. Furthermore, the proposed NSET algorithm yielded the highest performance for swollen neck classification with an mAP50 of 92.0%, significantly outperforming the baseline deep learning models while maintaining lower computational complexity.

## 1. Introduction

Hyperthyroidism-associated exophthalmos is a common endocrine and ophthalmic disorder that manifests through excessive thyroid hormone secretion and ocular abnormalities such as eyelid retraction, proptosis, and diplopia. Regarding conventional pattern-based diagnostic approaches, they rely heavily on clinical observations and the subjective experience of physicians in evaluating thyroid and ocular features, which often leads to inconsistency. Recent progress in artificial intelligence (AI) and deep learning has significantly advanced medical imaging diagnostics, offering quantitative and reproducible tools for disease identification.

For instance, the authors of [1] applied deep convolutional neural networks (CNNs) to thyroid scintigraphy images for automated diagnosis, achieving multicenter validation. Similarly, the deep learning-based eyelid morphology analysis [2] has been demonstrated to detect thyroid-associated ophthalmopathy with high accuracy. Clinical evaluation further emphasizes symptom-based categorization, yet this diagnostic process often lacks objective quantification. In [3], the authors reported a meta-analysis on the safety of propylthiouracil versus methimazole in hyperthyroid pregnancies, emphasizing the need to integrate medical imaging with precision medicine for improved strategies. While multi-parameter evaluations emphasize symptom-based categorization, these processes often lack objective quantification.

According to standardized assessments, AI-based assessment in [4] was developed to standardize clinical activity score (CAS) assessments and detect active thyroid eye disease (TED). Utilizing a ResNet-18 architecture, the machine learning (ML)-assisted workflow incorporates image preprocessing, server-side quality assurance, and multiple sign-specific classifiers.

By combining AI-detected inflammation with patient-reported pain, this system enables consistent early detection and remote monitoring. Similarly, a deep learning framework in [5] was developed for diagnosing thyroid eye disease, focusing on inflammation, eyelid retraction, and motility and further demonstrating machine learning’s role in integrated ophthalmic workflows. These approaches align with modern CNN-based frameworks, which have become the foundation for medical image analysis. CNNs effectively capture hierarchical spatial features, facilitating classification, detection, and segmentation tasks with exceptional efficiency and accuracy [6,7].

Furthermore, the multimodal medical image fusion in [8] has combined complementary information from multiple imaging modalities to enhance diagnostic reliability. To address data scarcity, self-supervised learning methods have also been adopted for medical image classification, improving generalization across domains [9]. Beyond thyroid imaging, deep learning has been extensively applied in other medical fields, demonstrating versatility and scalability. In [10], the CNN-based frameworks for arrhythmia classification were reviewed using electrocardiogram (ECG) signals, while the authors of [11] explored deep learning in terms of histopathology for predicting gene expression in breast cancer.

In ophthalmology, high-accuracy deep learning models were developed in [12] for diabetic retinopathy classification, highlighting AI’s value in visual assessment. In [13], further surveyed edge AI applications were applied in computer vision for real-time medical diagnostics by enabling deployment on portable and resource-limited devices. Lightweight deep learning architectures demonstrated efficient COVID-19 X-ray classification, providing a foundation for developing compact, high-accuracy diagnostic systems adaptable to thyroid and ocular imaging in [14]. Additionally, attention-based mechanisms improved model interpretability in [15] by enhancing diabetic retinopathy classification through a dual-attention deep learning model.

Therefore, this research article proposes an integrated diagnostic framework for facial and cervical image analysis to identify and classify hyperthyroidism-associated exophthalmos and thyroid enlargement. The framework mimics the biological visual mechanism by separately processing eye and neck regions, similar to how the human visual system analyzes different anatomical cues in parallel. These complementary features are then fused to form a unified diagnostic decision, resembling the brain’s integration of multi-source visual information. Leveraging a deep learning-based architecture, the proposed system utilizes face mesh landmarks for precise eye localisation. These localized regions are subsequently evaluated through two distinct methods: classification using the preprocessed YOLO model for exophthalmos detection and scleral area quantification via the proposed sclera map unwrapping engine (SMUE) algorithm. Simultaneously, for the cervical region, the system employs YOLO to identify the upper and lower neck segments, followed by an assessment of thyroid swelling using the neck μ−σ ensemble thresholding (NSET) algorithm. By integrating these three analytical components—deep learning-based eye classification, SMUE-based scleral analysis, and NSET-based cervical assessment—this framework provides a robust, standardized, and objective decision support tool, effectively enhancing early diagnosis and clinical evaluation of thyroid-related disorders.

## 2. Related Models and Backgrounds

Recent advancements in deep learning have significantly enhanced the diagnostic capabilities of medical imaging, particularly in the detection and classification of thyroid disorders. CNNs remain the foundation of automated image interpretation, enabling accurate extraction of structural and textural information from ultrasound images. In [16], the demonstration showed that CNN-based models can effectively distinguish benign from malignant thyroid nodules by underscoring the diagnostic value of convolutional architectures in the real-world clinical workflows.

Expanding on this foundation, researchers have explored more complex architectures as residual networks (ResNet). In [17], a ResNet18 framework was employed to enhance thyroid nodule assessment by leveraging residual connections to stabilize deeper feature learning. Meanwhile, a scoping review [18] of 13 studies to evaluate the efficacy of various AI models in diagnosing and assessing thyroid eye disease highlighted high diagnostic accuracy while identifying critical gaps in the study design and standardized reporting for clinical application. The development of lightweight and scalable networks has further accelerated progress toward deployable diagnostic systems. EfficientNet B0, with its compound scaling and parameter optimization, has emerged as an optimal model for medical imaging tasks that require both precision and efficiency.

Similarly, MobileNet and its successors, including MobileNetV2 and MobileNetV3 Small, utilize depthwise separable convolutions to achieve rapid inference with minimal computational resources. A lightweight CNN framework [19] was utilized to automate the assessment of thyroid eye disease (TED) severity from facial photographs, providing a non-invasive tool for evaluating clinical activity and severity levels with high diagnostic reliability, while the authors in [20] introduced a spatio-temporal cross-attention framework that analyzes cine thyroid ultrasound sequences, combining lightweight CNN backbones with temporal reasoning to capture dynamic glandular movement. In parallel, a neural network based on multi-scale feature fusion for differentiating thyroid follicular neoplasms [21] was developed by validating the effectiveness of deep multi-resolution learning in complex diagnostic contexts.

Dense connection architectures have become instrumental in the analysis of hyperthyroidism and TED due to their superior feature propagation and efficient gradient reuse. These structural advantages are particularly effective for extracting intricate pathological features from medical imaging, such as subtle extraocular muscle enlargement or periorbital tissue inflammation captured via clinical photographs or specialized scans. By facilitating deep feature integration, these models enhance the precision of disease severity grading and activity assessment. When integrated with ultra-efficient frameworks like MobileNetV2 and MobileNetV3, these architectures provide the necessary representational power to balance high-fidelity diagnostic accuracy with the computational feasibility required for modern clinical decision support systems. In [22], it was reported that DenseNet121 achieved superior results in analyzing dynamic ultrasound videos for predicting cervical lymph node metastasis in papillary thyroid carcinoma, and DenseNet169 [23] was identified as the top performer in a comparative study across multiple transfer learning methods for thyroid nodule classification.

Furthermore, recent advancements in image analysis, as demonstrated in [24], have emphasized the efficacy of utilizing statistical measures such as mean–std values to establish region-specific thresholds for decision making. By implementing a mean–std value per region criterion, it is possible to capture intricate details and subtle variations within localized areas of an image, facilitating a more robust and parameter-efficient workflow. This approach offers a significant advantage in enhancing classification accuracy without the need for extensive computational training, providing a reliable alternative for identifying complex physical characteristics even in scenarios with limited data availability. In [25], a comprehensive overview of AI-guided thyroid ultrasound segmentation and classification was provided by highlighting the growing convergence between deep convolutional architectures, attention mechanisms, and radiomics-informed models.

Regarding image-based analysis for thyroid classification, the authors of [26] recently introduced an automated framework utilizing a modified EfficientNetB2 model. The study leveraged a dataset of 7288 thyroid-related ultrasound images, demonstrating the efficacy of compound scaling and deep feature extraction in distinguishing between healthy states and thyroid conditions.

Additionally, thermal image analysis was employed in [27] as a diagnostic approach for hyperthyroidism. This study focused on characterizing temperature distribution patterns across three symptomatic regions: the eyes, neck, and shins. By utilizing thermal camera technology combined with digital image processing, the researchers performed a quantitative evaluation of physiological heat patterns. The findings demonstrate that localized thermal variations serve as reliable indicators of thyroid abnormalities, further supporting the validity of multi-region visual screening as a cost-effective alternative to conventional clinical methods.

Furthermore, the extreme learning machine algorithm in [28] was utilized to address noise issues in medical imaging, employing Wiener filtering for image enhancement prior to processing. One key contribution of this study is hyperparameter optimization, which significantly improves learning speeds and generalization capabilities. Tested on ultrasound datasets, the findings demonstrate the effectiveness of integrating rapid learning algorithms with metaheuristic optimization to achieve high diagnostic accuracy through computationally efficient methods.

Lastly, the authors of [29] introduced a hybrid diagnostic model that integrates deep learning with a support vector machine (SVM) classifier. This approach utilizes deep neural networks for robust feature extraction from thyroid datasets, subsequently employing an SVM for the final classification of hypothyroidism and hyperthyroidism. The combination of deep feature representations and the distinct marginal separation provided by SVMs establishes a highly reliable framework for automated thyroid diagnosis.

Collectively, these studies demonstrate how the integration of spatial, temporal, and contextual learning across architectures from lightweight models like CNNs and YOLOv11s into high-capacity models like DenseNet drives the evolution of intelligent thyroid image analysis. This convergence of computational efficiency, diagnostic accuracy, and clinical interpretability underscores the transition toward standardized, explainable, and accessible AI-assisted frameworks for precision thyroid disease evaluation.

## 3. Proposed Hyper-Thyro Vision Framework

The objective of this research is to establish an integrated framework that harmonizes pattern-based clinical knowledge with AI. By leveraging deep learning for the systematic differentiation and classification of diverse clinical syndromes, this framework facilitates the advancement of modern diagnostic instrumentation.

**Figure 1:**
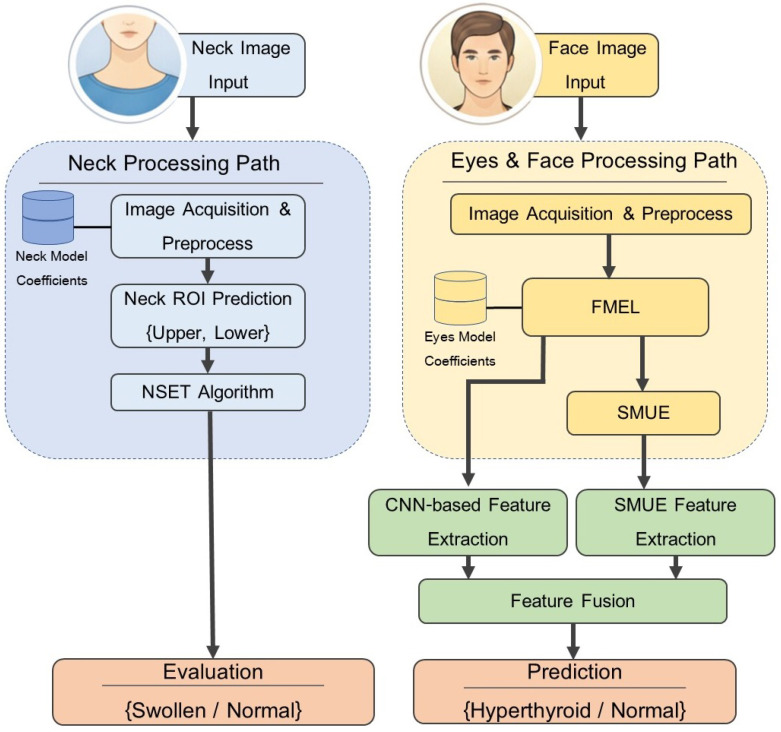
Hyper-Thyro Vision system architecture.

Figure 1 demonstrates the proposed system architecture, which is meticulously designed for health diagnostics through a dual-pathway image processing framework. The system initiates with two primary data sources: a neck image and a face image. Within the neck processing path, following image acquisition and initial preprocessing, the system utilizes the neck region of interest (ROI) prediction {upper, lower} module (guided by neck model coefficients) to precisely identify the region of interest and predict the positioning of upper and lower neck segments. This spatial information is then processed via the proposed NSET algorithm for a final clinical evaluation, classifying the condition as either swollen or normal.

The eyes and face processing path serves as the core component for high-dimensional feature extraction. Subsequent to initial preprocessing, the system employs the proposed face mesh-based eye landmark (FMEL) algorithm integrated with eye model coefficients to extract both eye regions from the facial image and perform an eyes unfold operation. This transformation converts the ocular geometry into a flattened representation suitable for advanced analysis. These data are then fed into the proposed sclera map unwrapping engine (SMUE) to calculate the sclera metrics, effectively generating distinct feature sets derived from both the left and right eye characteristics.

In the final analytical stage, the framework adopts a dual-branch architecture for comprehensive feature representation. The first branch executes CNN-based feature extraction to capture intricate visual embeddings directly from the unfolded eye images. Simultaneously, the second branch performs SMUE feature extraction to derive embeddings from the quantitative statistical metrics obtained earlier. These heterogeneous feature vectors are integrated through a feature fusion layer, merging spatial visual patterns with localized statistical data. The fused embedding is then passed to the final prediction layer to classify the patient’s diagnostic result as either hyperthyroid or normal. This multi-modal approach significantly enhances the system’s robustness by leveraging the synergy between visual evidence and quantitative clinical indicators.

**Figure 2:**
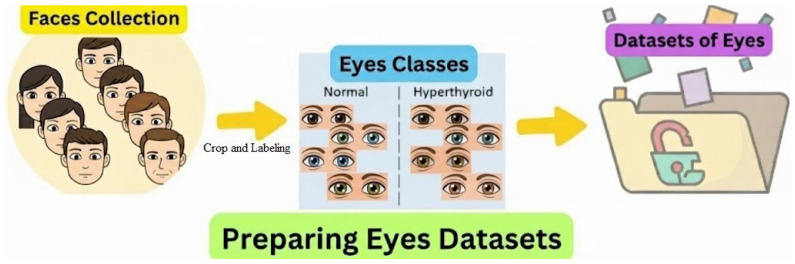
Workflow for preparing the eye image dataset from face collection to cropping, labeling, and classification into normal and hyperthyroid classes.

The overall workflow for preparing the eye dataset is illustrated in Figure 2. This process involves three sequential stages before the images are stored for subsequent training and evaluation: face collection for initial acquisition of raw facial images, cropping and labeling for precise isolation, and annotation of the bilateral eye regions. Classification assigns each image to one of two distinct clinical classes: normal or hyperthyroid.

**Figure 3:**
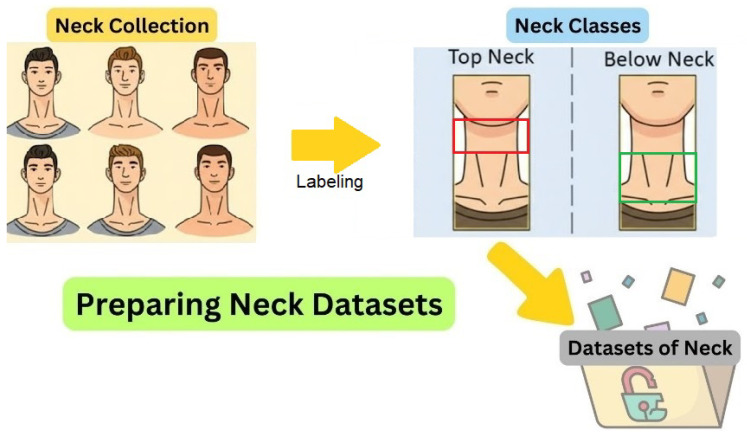
Workflow for preparing the neck image dataset, from image collection to labeling and classification into neck, top neck, and below neck classes.

Another essential component is the preparation of the neck image dataset. The neck region often reflects clinical abnormalities associated with thyroid-related disorders, such as goiters or localized swelling. Therefore, the collection and classification of neck images were specifically designed to enable the model to effectively learn and distinguish between these different physical characteristics. As illustrated in Figure 3, the workflow for preparing the neck dataset begins with neck collection, where images are captured while focusing exclusively on the cervical region. These collected images are then subjected to a labeling process, in which bounding boxes are assigned to define specific regions of interest (ROIs).

The labeling categorizes the data into two distinct classes: “top neck”, representing the upper portion of the neck, and “below neck”, representing the lower portion. Both of these segments are subsequently utilized for further analysis of swelling symptoms. After labeling, all images are stored in the neck dataset, which serves as the foundation for model training and evaluation. Following the preparation of eye and neck image datasets, the next stage of the proposed system is the training process, which is crucial for developing models capable of accurate clinical prediction and classification. In this step, the preprocessed and labeled datasets are systematically partitioned into training and testing subsets, enabling rigorous learning and performance evaluation of the models.

**Figure 4:**
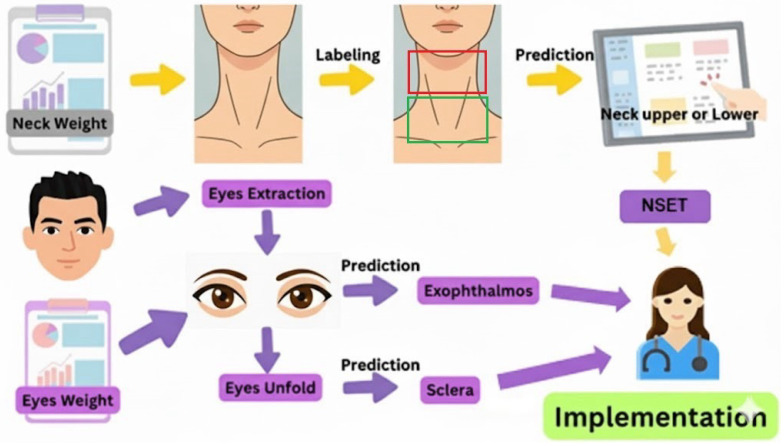
Training process of the proposed system using neck and eye datasets, divided into training and testing subsets for learning and validation.

As illustrated in Figure 4, the training process is performed separately for the neck dataset and the eyes dataset. For each dataset, a training set is used for the iterative learning phase. A test set is reserved for an unbiased evaluation of model performance. Upon completion of the training phase, the models yield their learned parameters, referred to as the neck weight and eyes weight. These weights represent the final trained models ready for subsequent prediction and analysis tasks within the integrated framework. After successfully training the models with the neck and eye datasets, the subsequent stage is implementation.

In this phase, the trained model weights, namely the neck weight and eyes weight, are applied to new, real-world images to evaluate the system’s capability for predicting clinical features associated with thyroid-related disorders. The proposed system utilizes these learned parameters to process new input images and generate predictions for both the neck and eye regions of interest. This final step validates the diagnostic utility and predictive accuracy of the integrated framework.

### 3.1. Source of Data and Collection

In this study, a systematic literature search was conducted to identify relevant publications, including clinical practice guidelines and expert consensus documents focused on integrated clinical pattern recognition. This identification was achieved through systematic literature searches across various international and regional academic databases, including PubMed and other pertinent platforms. Regarding the data collection process, the diagnostic criteria were established by analyzing abnormalities in the ocular and cervical regions. For the eye dataset, the research analyzed a total of 230 images, consisting of 130 images of healthy eyes sourced from a public dataset and 100 images of patients exhibiting thyroid-related ocular abnormalities. These patient images were specifically collected as a private dataset for this study. The cohort was meticulously controlled for demographic diversity, including 157 females and 73 males across various age groups to minimize potential confounding variables.

Similarly, the neck dataset focused on identifying the characteristics of goiters or thyroid enlargement, with data categorized into normal and swollen classes. All images in the swollen category were obtained from a private dataset to ensure clinical relevance. The labeling and verification processes for both the eye and neck private datasets were conducted under the direct supervision of medical experts to ensure the highest level of clinical accuracy for the multi-modal diagnostic framework.

### 3.2. Diagnostic Criteria

Facial images were analyzed using a face mesh detection approach implemented with MediaPipe FaceMesh to obtain a dense set of facial landmarks that accurately described the periocular geometry. In our pipeline, each image was processed independently in single-image mode to avoid reliance on temporal tracking, and the model was restricted to one face per image to ensure deterministic landmark selection when multiple faces might appear. Refined landmark estimation was enabled to improve landmark stability around fine structures, which is critical for eye-related analysis, where small localisation errors can propagate into downstream measurements. The face mesh module returns landmarks in normalized coordinates relative to the image size; therefore, the landmark locations were converted into pixel coordinates to enable precise geometric operations in the original image coordinate system. If no landmarks were returned due to, for example, severe occlusion, extreme head poses, motion blur, or inadequate face visibility, then the sample was treated as a detection failure and excluded from subsequent eye region extraction to prevent generating incorrect regions that could introduce noise into the learning and measurement stages.

To define the ROIs, periocular anchor landmarks corresponding to the eye corner (canthus) region were selected for both eyes, because these points are anatomically stable and provide consistent reference locations across subjects. In our implementation, two landmarks were used per eye to form robust anchor pairs (left eye: indices 33 and 133; right eye: indices 362 and 263), yielding four reference points that reliably spanned the horizontal extent of the periocular region. Using the pixel coordinates of these points, the system computed an initial bounding region that covered both eyes by taking the minimum and maximum landmark coordinates in the horizontal and vertical directions, ensuring that the ROI always enclosed the eye span even under moderate inter-subject variability. Because diagnostic eyelid assessment requires full visibility of the eyelid contours and scleral exposure rather than only the eye corners, the initial bounding region was expanded using fixed padding margins on both axes.

This padding serves two practical purposes. (1) It retains clinically relevant surrounding tissue (upper and lower eyelids and adjacent periocular skin) that contributes to eyelid retraction and scleral area analysis. (2) It increases robustness against minor landmark jitter and small pose differences. Finally, all ROI boundaries were clamped to the valid image range to prevent out-of-frame cropping and to guarantee that the ROI extraction remained valid across the full dataset.

Eye extraction was then performed by cropping the finalized ROI directly from the original facial photograph to generate a standardized periocular image for subsequent analysis. This extraction step reduces input dimensionality and removes non-target facial components (e.g., cheeks, nose, hair, and background) that are irrelevant to eye-based diagnosis, thereby improving the signal-to-noise ratio for the learning model and measurement routines. By consistently applying the same cropping rules across all baseline and follow-up images, the extracted eye ROIs maintain a comparable anatomical framing, which improves reproducibility and helps ensure that observed longitudinal changes reflect true morphological progression rather than differences in camera framing or irrelevant facial variation. The resulting eye region images were saved as the primary inputs for downstream deep learning and automated quantification of eyelid morphology, supporting more reliable detection of features such as eyelid retraction patterns, palpebral fissure characteristics, scleral exposure changes, and periocular contour asymmetry.

For example, the model’s focus is on crucial parameters such as the eyelid aperture width, pupil size, and scleral area. The extracted eye images are then stored in the image dataset, which serves as the source for model training in subsequent stages. Each image in this dataset is annotated with either clinical labels or dimensional parameters derived from image-based computations. These dimensional parameters can be calculated according to the following equations:

(1) L=FaceMesh(I),

(2) $$\left(x_{i}^{\left(n\right)},y_{i}^{\left(n\right)}\right)=l_{i}\in L,\forall_{i}\in \varepsilon \;.$$

Let the facial image be denoted by *I*, and let *L* represent the set of normalized landmark points, where $x_{i}\left(n\right),y_{i}\left(n\right)\in \left[0,1\right].$ For this study, the landmarks were associated with the eye region. The actual pixel coordinates $\left(x_{i},y_{i}\right)$ of each landmark point i∈ε are then computed, where *W* and *H* denote the width and height of the image, respectively. Thus, the transformation from normalized coordinates to pixel coordinates can be expressed as follows:

(3) $$x_{i}=\left\lfloor x_{i}^{n}\cdot W\right\rfloor \;,\;\;y_{i}=\left\lfloor y_{i}^{n}\cdot H\right\rfloor \;.$$

Subsequently, the bounding box of the eye region is calculated and defined with an added margin *m*, This margin is specifically introduced to expand the bounding box around the detected landmarks, thereby ensuring that the entire relevant ocular region is fully captured for subsequent analysis. The process of calculating the bounding box and applying the margin is essential for accurately isolating the region of interest for model training. The margin is defined as a positive integer such that $m\in Z^{+}$.

The bounding box coordinates can therefore be expressed as follows:

(4) $$x_{min}=\max\left(0,\min_{i\in \varepsilon }x_{i}-m\right)\;,$$

(5) $$x_{max}=\min\left(W,\max_{i\in \varepsilon }x_{i}+m\right)\;,$$

(6) $$y_{min}=\max\left(0,\min_{i\in \varepsilon }y_{i}-m\right)\;,$$

(7) $$y_{max}=\min\left(H,\max_{i\in \varepsilon }y_{i}+m\right)\;.$$

Finally, the eye region is cropped from the original image based on the bounding box defined by Equation (4) through Equation (7). This operation can be expressed as follows:

(8) $$I_{eye}=I\left[y_{min}:y_{max}\;,\;x_{min}:x_{max}\right].$$

**Figure 5:**
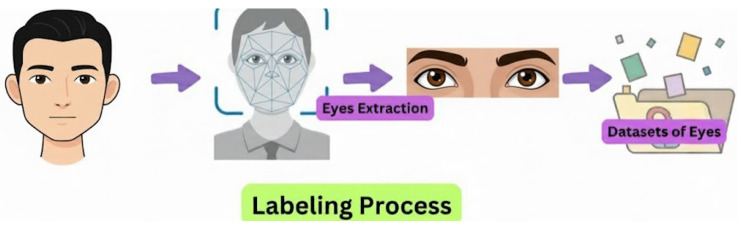
Workflow for eye region labeling.

**Figure 6:**
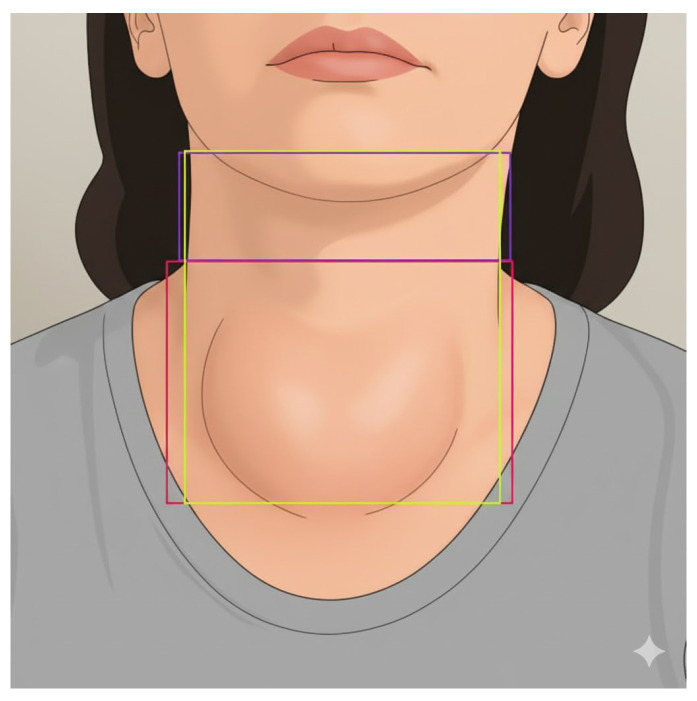
Workflow for neck region labeling.

The overall workflow of proposed FMEL for extracting the eye region, which serves as the input for subsequent training process, can be formally summarized in Algorithm 1. It formalizes the robust pipeline for transforming raw facial imagery into high-fidelity periocular segments optimized for the subsequent training of deep learning diagnostic models. For the neck region, data collection was performed by capturing images focusing exclusively on the cervical area. Since these images already isolated the region of interest, they could be directly stored in the database and annotated with bounding boxes, as illustrated in Figure 5. The bounding box annotations were meticulously divided into three distinct classes, as shown in Figure 6: neck, top neck, and below neck. This direct approach ensured that the annotation accurately reflected the anatomical regions required for subsequent deep learning analysis.
**Algorithm 1:** Face mesh-based eye landmark (FMEL).***Input**: Input face image (I)****Output**: Cropped eye region image ($I_{eye}$)****Initial of variables**: assign image width and height to variables w and h; define**    $LEFT_{EYE}=\left[33,133\right],RIGHT_{EYE}=\left[362,263\right]$; initialize empty list $eye_{points}$** *   1   *Process I using FaceMesh model to results*   2   ***if** results contains face landmarks **then***   3         ***for each** index i in $LEFT_{EYE}$ ∪ $RIGHT_{EYE}$ **do***   4              $x_{i}=int\left(landmark\left[i\right].x*w\right)$   5              $y_{i}=int\left(landmark\left[i\right].y*h\right)$   6             ***append** ($x_{i},y_{i}$) to eye_points*   7         ***end for***   8         $x_{min}=\min\left(x_{i}\right)-50;\;x_{max}=\max\left(x_{i}\right)+50$   9         $y_{min}=\min\left(y_{i}\right)-50;\;y_{max}=\max\left(y_{i}\right)+50$   10       *clip $x_{min},x_{max}$ **within** [0, w]*   11       *clip $y_{min},y_{max}$ **within** [0, h]*   12       $I_{eye}$ = ***crop image** I **from*** $\left(x_{min},y_{min}\right)$ to $\left(x_{min},y_{min}\right)$   13   ***end if***

### 3.3. Eye and Neck Prediction Process

The process of forecasting clinical characteristics from the eye and neck regions is an artificial intelligence–based procedure designed to detect and predict relevant clinical outcomes. This process can be formulated mathematically as follows:

(9) $$I_{eye}=Crop\left(I_{face}\right)\;.$$

Let $I_{eye}$ denote the extracted eye region obtained from the facial image $I_{face}$ using Algorithm 1. The resulting image $I_{eye}$ is then used as input to the eye prediction model $M_{eye}$. This can be expressed by

(10) $$result_{eye}=M_{eye}\;\left(I_{eye}\right)\;.$$

For the neck prediction process, the model $M_{neck}$ is employed, where $I_{neck}$ denotes the neck image of the subject. The prediction can be expressed as follows:

(11) $$result_{neck}=M_{neck}\;\left(I_{neck}\right)\;.$$

Therefore, to systematically classify images based on the morphological biomarkers associated with thyroid-related pathologies such as periocular protrusion in exophthalmos and cervical hypertrophy indicative of goiters, this process employs the Hyper-Thyro Vision framework. This framework executes a multi-stage computational pipeline grounded in the integration of advanced computer vision heuristics and clinical diagnostic criteria, providing a rigorous methodology for the automated identification and symptomatic categorization of thyroid disorders as detailed in Algorithm 2.
**Algorithm 2:** Hyper-Thyro Vision framework.***Input**: Input face image ($I_{face}$)* and *Cropped eye region image ($I_{neck}$)****Output**: Clinical prediction label for eye and neck****Initial of variables**: Load Eye Model* $M_{eye}$ and Neck Model $M_{neck}$. Set confidence threshold *t* and initialize result logR* *    1   ***Load** full-face image $I_{face}$*    2   ***Detect** facial landmarks **using** Face Mesh*    3   ***Perform inference:***       $result_{eye}=M_{eye}\left(ROI_{eye}\right)$    4   ***If** $result_{eye}$ **contains** label “HYPERTHYROID”*    5   ***with** confidence* ≥ *t*:    6    $Label_{eye}=“HYPERTHYROID”$    7   ***Else:***    8    $Label_{eye}=“Not\;HYPERTHYROID”$    9   ***Load** neck image* $I_{neck}$    10  ***Perform inference:***      $result_{neck}=M_{neck}\left(I_{neck}\right)$    11  ***If** $result_{neck}$ **contains** label with confidence* ≥i:    12    $Label_{neck}=predictedclassfromresult_{neck}$    13   ***Else:***    14    $Label_{neck}=“UNCERTAIN”$    15  ***Record** prediction results:*     $R=[Label_{eye},Label_{neck}]$    16   ***Return R***

### 3.4. Eye Unfold and Calculation Sclera Area

To improve the accuracy of the model in detecting ocular abnormalities such as thyroid-associated exophthalmos, this study developed a specialized data preparation procedure referred to as the eye unfold technique. This process involves extracting and transforming the scleral region of the eye with the aim of enhancing the system’s capability to analyze the morphological characteristics of the eyes more effectively and in a standardized manner. The standardization minimizes variations caused by the three-dimensional curvature of the eyeball, thereby improving the consistency and reliability of subsequent deep learning analysis.

**Figure 7:**
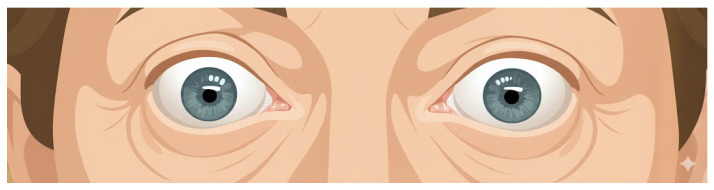
Example of cropped image results obtained using positions derived from face mesh detection.

The workflow of the eye unfold process begins with the detection of facial landmarks using the face mesh model, which enables the identification of precise coordinates for cropping ROIs from a patient’s full-face image, as illustrated in Figure 7. The resulting cropped image is then processed to focus primarily on the scleral region.

At this stage, the eye unfold procedure takes this cropped eye image, denoted as $I_{eye}$, and the procedure then performs the computations emphasizing the scleral region. The process involves determining the pupil center based on Equations (12)–(14).

The centroid coordinates of the eyes are then calculated, yielding $c_{x}$ and $c_{y}$, and the set of centroid positions is denoted collectively as *c*:

(12) $$m_{pq}=\sum_{(x,y)\in C}x^{p}y^{q}\;,$$

(13) $$\left(c_{x},c_{y}\right)=\left(\frac{m_{10}}{m_{00}},\frac{m_{01}}{m_{00}}\right)\;,$$

(14) $$c=(c_{x},c_{y})\;,$$

(15) $$D_{max}=\max_{i}∥C_{i}-c∥\;,$$

(16) $$R_{max}=\lceil D_{max}+\beta \rceil \;.$$

It is important to note that Equations (12)–(16) are applied to both the left and right eyes independently. Using the landmark data derived from the proposed FMEL algorithm, the boundaries of two eyes {left, right} are obtained, with each landmark position represented by *C*.

The next step involves determining the radius for the unfolding process by calculating the maximum distance from the pupil’s center to the eyelid boundary, as formally expressed in Equation (15). This radius value is crucial, as it defines the spatial extent of the scleral region that will be transformed and standardized during the unfolding procedure.

The overall maximum distance, denoted as $D_{max}$, is then used to establish the maximum eye unfolding radius $R_{max}$ according to Equation (16). Here, β represents a constant that serves as an adjustment factor, providing flexibility for the unfolding process in practical applications.

**Figure 8:**
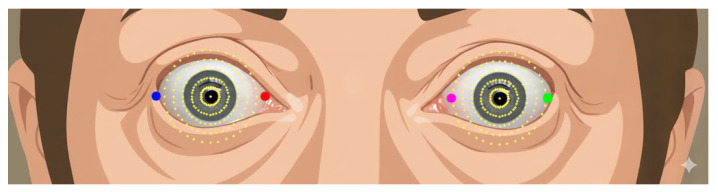
Radial positions for the eye unfolding process in the left and right eyes, derived from face mesh landmarks and geometry. Red dots represent boundary landmarks, green dots indicate center reference points, blue dots denote sampling positions, and pink dots represent auxiliary reference landmarks used for geometric alignment.

The positions searched to identify the boundaries of the left and right eyes can be illustrated in a circular representation, as shown in Figure 8. Once the radius for the unfolding process has been determined, the eye images can be unfolded by

(17) $$r_{i}=\frac{i}{H-1}R_{max}\;,\;\;\theta_{j}=\frac{j}{W-1}\pi$$

(18) $$x_{ij}=c_{x}+r_{i}\cos\theta_{j}\;,\;\;y_{ij}=c_{y}+r_{i}\sin\theta_{j}$$

(19) $$U(i,j)=\left(I_{eye};\left(x_{ij},y_{ij}\right)\right).$$

Here, (i,j) denote the pixel positions along the vertical and horizontal axes, while *H* and *W* represent the height and width of the eye region, respectively. The unfolding radius is expressed as $r_{i}$, and the angular resolution of the unfolding is represented by $\theta_{j}$. The final unfolded eye image is denoted by U(i,j).

**Figure 9:**
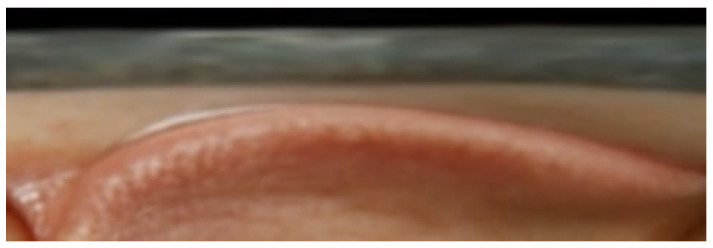
Left eye unwrapped.

**Figure 10:**
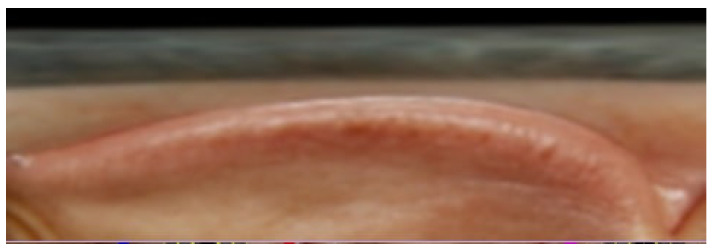
Right eye unwrapped.

From the eye unfold method, the resulting standardized images are obtained, as shown in Figure 9 and Figure 10, which respectively illustrate the computed unfolded images for the left and right eyes, respectively. The subsequent process of examining the scleral region involves reading along the eye boundary by traversing the eyelid contour.

The measurement procedure formally begins by defining the positions and distances for scleral sampling, as expressed below:

(20) $$r=\max(\sqrt{\Delta x^{2}+\Delta y^{2}})$$

(21) $$\left(\Delta x,\Delta y\right)=(x-c_{x},y-c_{y}).$$

Here, *r* represents the distance from the pupil’s center, with an offset (Δx,Δy) calculated from the eyelid boundary points (x,y) relative to the eye centroid $\left(c_{x},c_{y}\right)$.

Subsequently, the calculation proceeds by determining the semicircular angle in order to obtain the coordinates around the entire eye prior to unfolding. This is computed by

(22) $$\theta_{1/2}=\arccos\left(\frac{\Delta x}{r}\right)\in \left[0,\pi \right]$$

(23) $$\left(u,v\right)=\left(\frac{\theta_{1/2}}{\pi }\left(W-1\right),\min\left(\frac{r}{R_{max}}\right)\left(H-1\right)\right)$$

(24) $$UV=\{\left(u_{j},v_{j}\right)\}.$$

where $\theta_{1/2}$ denotes the semicircular angle corresponding to the upper or lower eyelid boundary. The coordinates (u,v) represent the calculated positions along the upper and lower eyelids, respectively, and all positions are aggregated and denoted as UV.

**Figure 11:**
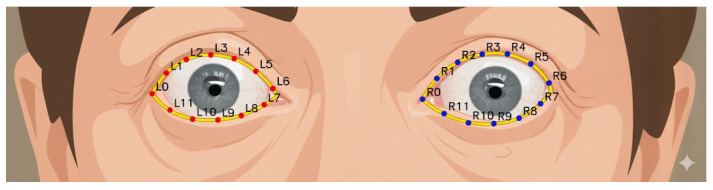
Eyelid curves.

These coordinates allow identification of the eye boundary and are visualized in Figure 11, which illustrates sequentially mapped positions along the upper eyelid, eye corners, lower eyelid, and lower eyelid margin. The left eye is indexed as {L0–L11}, while the right eye is indexed as {R0–R11}. These positions serve as reference points for subsequent scleral distance measurements.

**Figure 12:**
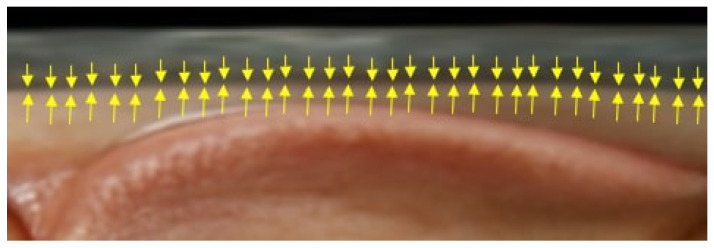
Scleral boundary determination, indicated by yellow arrows from the eyelid margin to the iris edge, used as the endpoint for pixel-based measurement.

As illustrated in Figure 12, the arrows indicate the terminal boundary of the sclera, extending from the eyelid margin to the iris pattern. This location is defined as the end point for vertical pixel-based measurements starting from the eyelid edge. Identifying this boundary is critical for determining the effective scleral dimension at each position, as it represents the true anatomical limit of the sclera.

Once accurately detected, these pixel distances can be quantitatively analyzed to evaluate the scleral size across different regions. This forms a fundamental step in assessing ocular abnormalities such as thyroid-associated exophthalmos. The final step involves measuring the scleral distances at positions {L0–L11} and {R0–R11}.

To simplify this measurement process, the algorithm employs the unfolded eye image, which allows the scleral regions at each starting position to be measured in a straight-line manner toward the pupil boundary. Based on the previous equations, the starting points of the measurements from the eye boundary are already defined.

In the proposed algorithm, the endpoint is defined as the onset of iris pattern, which is precisely located by

(25) $$\left(\mu_{above}\left(y\right),\mu_{below}\left(y\right))=(L\left(y-\omega_{a}\right),L\left(y+\omega_{b}\right)\right)$$

(26) $$y^{*}=\arg\max_{y}(\mu_{above}\left(y\right),\mu_{below}\left(y\right))$$

(27) $$d_{j}=\;|v_{j}-y^{*}|$$

(28) $$D=[d_{0},\ldots ,d_{K-1}].$$

This detection is achieved by finding the point of maximum intensity variation between the sclera and the iris. This is calculated by comparing the pixel intensity values at the previous and subsequent positions along the y axis, denoted as $\mu_{above}\left(y\right)$ and $\mu_{below}\left(y\right)$, respectively. The position of maximum change is defined as $y^{*}$. This process is repeated for all measurement directions, resulting in distances represented by $d_{j}$, with the complete set of results for the left eye indexed as {L0–L11} and that for the right eye indexed as {R0–R11}, collectively denoted as *D*.

**Figure 13:**
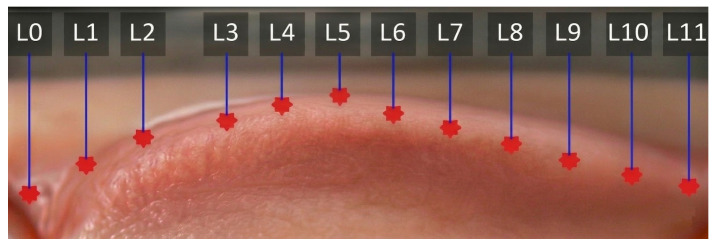
Left eye unwrapped with {L0–L11}.

**Figure 14:**
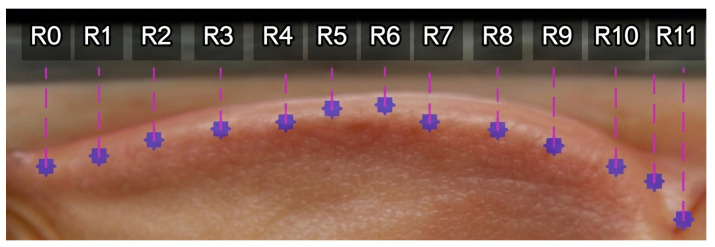
Right eye unwrapped with {R0–R11}.

The overall results of the computation allow for the measurement of scleral distances, as illustrated in Figure 13, which shows the vertical scleral distance measurements for the left eye from position L0 to L11. Similarly, Figure 14 presents the vertical scleral distance measurements for the right eye from position R0 to R11.

The sclera-map unwrapping engine (SMUE) constitutes a fundamental computational component of the ocular predictive architecture, as formalized in Algorithm 3. This engine orchestrates a systematic sequence of geometric transformations and nonlinear mapping to transpose the curved ocular surface into a flattened representation. By integrating automated feature localisation with morphological image processing, the engine enables the precise segmentation and quantification of scleral boundaries, providing the high-fidelity spatial data requisite for determining critical scleral dimensions in clinical analysis.
**Algorithm 3:** Sclera map unwrapping engine (SMUE).***Input**: Input eye image ($I_{eye}$), unwrap size(H, W)****Output**: Set of sclera lengths ($D_{0,1,\ldots ,11}^{L}$, $D_{0,1,\ldots ,11}^{R}$ )****Initial of variables**: -** *   1   ***Load** $I_{eye}$ and Get eye landmarks*   2   ***For each** eye {Left, Right}*   3   ***Find** pupil center*   4   ***Find** $D_{max}$ and $R_{max}$*   5   ***Unwrap** from $I_{eye}$ for i = 0, …, H-1, j = 0, …, W-1*   6     ***Find** r, θ and UV*   7   ***Map** points to UV={{L0−L11},{R0−R11}}*   8   ***For each** UV*   9     ***Calculate** $L,\mu_{above},\mu_{below}$*   10       $y^{*}=\arg\max(\mu_{above}\left(y\right),\mu_{below}\left(y\right))$   11       ($D_{0,1,\ldots ,11}^{L}$, $D_{0,1,\ldots ,11}^{R}$) = ***Obtain** sclera lengths from* $y^{*}$   12   ***Return*** ($D_{0,1,\ldots ,11}^{L}$, $D_{0,1,\ldots ,11}^{R}$)

### 3.5. Neck μ−σ Ensemble Thresholding (NSET)

**Figure 15:**
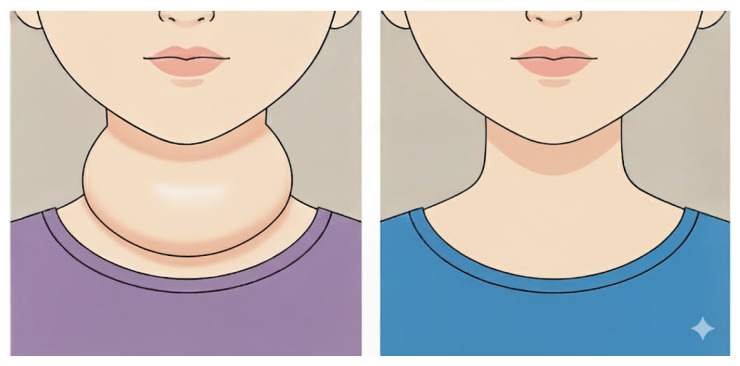
(**left**) Example of a swollen neck caused by thyroid enlargement. (**right**) Example of a normal neck.

The analysis of images exhibiting swollen neck characteristics employs measurement criteria derived from the image processing of the three-deep learning-obtained neck-related classes: neck, top neck, and below neck. These processed outputs are subsequently used to evaluate swelling in the cervical region. Since the dataset of neck images from patients with swelling symptoms is limited in size and thus insufficient to enable highly accurate deep learning-based prediction, this study introduces an image analysis procedure specifically designed to assess neck swelling. The approach emphasizes visual differentiation of the swelling characteristics as illustrated in Figure 15:

(29) $$\left(C_{T},C_{L}\right)=\left(center(T),center(L)\right)\;.$$

To prevent image rotation and normalize all images onto a consistent axis, it is necessary to first determine the orientation and required degree of rotation. This crucial preprocessing step ensures that anatomical features are consistently aligned across the entire dataset, thereby maximizing the robustness and accuracy of the subsequent deep learning analysis. The rotation degree and the corresponding transformation can be formally expressed as follows:

(30) $$\left(\vec{v},\phi \right)=\left(\left(C_{L}-C_{T}\right),\mathrm{atan}2\left(v_{y},v_{x}\right)\right)$$

(31) $$I^{\prime }=RotateScale(I,-\phi )\;.$$

The subsequent step involves standardizing the color of the neck surface region to eliminate variations in brightness, achieved through a normalization method such as contrast-limited adaptive histogram equalization (CLAHE), and a mask (M) is then generated to filter the region of interest as formulated below:

(32) $$I^{\prime \prime }=CLAHE(I^{\prime })$$

(33) $$M=PostProcess(SkinThresh\left(I^{\prime \prime }\right))\;.$$

This process employs basic operations such as skin color-based filtering combined with morphological opening–closing algorithms. The resulting enhanced image is denoted as *M*.

Next, the definitions for calculation of the evaluation metrics are established by first determining the neck width at each vertical level, as expressed by

(34) $$(x_{L}\left(y\right),x_{R}\left(y\right))=\left(\begin{array}{c} \min\{x|M(x,y)=1\}\;,\\ \max\{x|M(x,y)=1\} \end{array}\right)$$

(35) $$(W\left(y\right),W_{L}\left(y\right),W_{R}\left(y\right))=\left(\begin{array}{c} x_{R}\left(y\right)-x_{L}\left(y\right)+1\\ x_{m}-x_{L}\left(y\right),x_{R}\left(y\right)-x_{m} \end{array}\right)$$

The vertical axis is normalized such that y∈[0,1], representing positions from the top (‘0’) to the bottom (‘1’). Let $x_{m}$ denote the midpoint coordinate along the x axis in the aligned image. At each level $y,(x_{L}\left(y\right),x_{R}\left(y\right))$ correspond to the left and right boundaries, respectively, while $\left(W\left(y\right),W_{L}\left(y\right),W_{R}\left(y\right)\right)$ represent the total width, the left side’s width, and the right side’s width, respectively.

The average widths of the upper and lower neck regions are then calculated by Equation (36), while the baseline linear measurement representing the characteristics of a normal neck is determined using Equation (37):

(36) $$(W_{top},W_{bot})=\left(\begin{array}{c} \mathrm{mean}\{W(y):y\in [0.10,0.20]\}\;,\\ \mathrm{mean}\{W(y):y\in [0.80,0.90]\} \end{array}\right)$$

(37) $$W_{lin}=(1-y)\;W(0)+y\;W(1)\;.$$

Subsequently, four quantitative indicators are defined to evaluate the neck images: the top-to-low width ratio (TLR), bulge peak index (BPI), bulge area ratio (BAR), and asymmetry ratio (ASR). These metrics are used to characterize and assess the neck morphology and are formulated in Equations (38)–(41):

(38) $$TLR=\frac{W_{top}}{W_{bot}}$$

(39) $$BPI=\frac{max_{y}max\left\{W\left(y\right)-W_{lin}\left(y\right),0\right\}}{W_{bot}}$$

(40) $$BAR=\frac{1}{W_{bot}}\cdot mean_{y}\left(max\left\{W\left(y\right)-W_{lin}\left(y\right),0\right\}\right)$$

(41) $$ASR=\frac{\sum_{y}\left|W_{L}\left(y\right)-W_{R}\left(y\right)\right|}{\sum_{y}\left|W_{L}\left(y\right)+W_{R}\left(y\right)\right|}\;.$$

The final step is the decision-making process, using the μ−σ ensemble thresholding (NSET) as defined by

(42) $$\theta_{m}=\mu_{m}+k\;\sigma_{m}\;,$$

where the metrics m∈{TLR,BPI,BAR,ASR} are evaluated and where $\mu_{m}$ and $k\;\sigma_{m}$ represent the mean and standard deviation of the metric *m*, respectively, while *k* is a constant parameter.

Thus, the evaluation can be determined based on the number of metrics exceeding the defined thresholds derived from the four indicators, with the decision-making process formulated as

(43) $$f_{m}=\left\{\begin{array}{c} 1,\mathrm{if}\;\;m>\theta_{m}\\ 0,\mathrm{otherwise} \end{array}\right.$$

(44) $$\mathrm{score}=\sum_{m}f_{m}$$

(45) $$result=\left\{\begin{array}{c} Swollen,\mathrm{if}\;\;\mathrm{score}\geq q\\ Normal,\mathrm{if}\;\;\mathrm{score}<q \end{array}\right.$$

Here, *q* denotes the minimum number of metrics specified by the user as a criterion for classification.

In the NSET algorithm, μ and σ come from a normal (non-swollen) reference set. For each metric (TLR, BPI, BAR, and ASR), we first measure its values on many normal neck images. From those values, we compute the sample mean (μ) to represent the typical level for the normals and the sample standard deviation (σ) to represent natural variability around that level. These two statistics define a data-driven threshold for each metric, where values notably above the normal mean relative to its variability are treated as atypical, and a vote for swelling is given. In practice, μ and σ should be refreshed whenever imaging conditions, devices, or the target population change. The decision-making process using the NSET algorithm can therefore be expressed as shown in Algorithm 4.
**Algorithm 4:** Neck μ−σ ensemble thresholding (NSET) calculation.***Input**: T, L, N = YOLO boxes: Top-Neck, Low-Neck****Output**: result****Initial of variables**: k = 2.0, q = 2, S = 60, margin = 0.10** *   1   ***Obtain image** t, L and N from YOLO*   2   ***Alignment and find ROI** T and L*   3   *C_T_ = center(T)*   4   *C_L_ = center(L)*   5   *ν = C_L_-C_T_*   6   *phi = atan2(vy, vx)*   7   $s=L_{ref}/∥v∥$   8   $I^{\prime }={rotate}_{scale}\left(I,-phi,s\right)$   9   $ROI=expand_{union}\left(T,L,margin\right)$   10   ***Build neck mask***   11    *ROI’ = crop I’ by ROI*   12    $M=skin_{mask}\left(ROI^{\prime }\right)$   13   ***Width profile & metrics***   14    *x_m_ → horizontal midline x in ROI’*   15    *{W[0 … S-1], WL[0 … S-1], WR[0 … S-1]} = empty*   16    ***for** i = 0 … S-1:*   17    y = round(i*(height(ROI’)-1)/(S-1))   18    $\left(x_{L},x_{R}\right)=width_{at}\left(M,y\right)$   19    *if no neck pixels at row r: continue*   20    *W[i] = x_R_ - x_L_ + 1*   21    *WL[i] = x_m_ - x_L_*   22    *WR[i] = x_R_ - x_m_*   23    $top_{a}=round\left(0.10*\left(S-1\right)\right);top_{b}=round\left(0.20*\left(S-1\right)\right)$   24    $bot_{a}=round\left(0.80*\left(S-1\right)\right);bot_{b}=round\left(0.90*\left(S-1\right)\right)$   25    $W_{top}=mean_{range}\left(W,top_{a},top_{b}\right)$   26    $W_{bot}=mean_{range}\left(W,bot_{a},bot_{b}\right)$   27    ***if** W_bot_ ≤ 0: W_bot_ = 1*   28    ***for** i = 0 … S-1:*   29    W_lin_[i] = (1 - i / (S-1))* W[0] + (i / (S-1))*W[S-1]   30    *dev[i] = max(W[i] - W_lin_[i], 0)*   31    *TLR = W_top_ / W_bot_*   32    *BPI = max(dev) / W_bot_*   33    *BAR = mean(dev) / W_bot_*   34    *ASR = (∑ |W_L_[i] - W_R_[i]|) / (∑ (W_L_[i] + W_R_[i]))*   35   ***Decision: μ-σ Ensemble (NSET)***   36    ***for** m ∈ {TLR, BPI, BAR, ASR }:*   37    $\theta_{m}=\mu \left[m\right]+k*\sigma \left[m\right]$   38    *f[m] = 1 if value(m) > $\theta_{m}$ else 0*   39    *score = f[TLR] + f[BPI] + f[BAR] + f[ASR]*   40    ***if** score ≥ q: result = “Swollen” else “Normal”*   41   ***Return** result*

### 3.6. Performance Evaluation Criteria

To assess the effectiveness of the proposed framework, we employed a set of well-established evaluation metrics that are widely used in object detection tasks. These included accuracy, recall, and precision, which collectively provided a comprehensive view of the model’s performance. The fundamental metrics for overall correctness, reliability, and sensitivity are defined as follows:

(46) $$Accuracy=\frac{TP+TN}{TP+FN+TN+FP}$$

(47) $$Recall=\frac{TP}{TP+FN}$$

(48) $$Precision=\frac{TP}{TP+FP}$$

where TP (true positive) represents the number of correctly detected objects, TN (true negative) denotes the number of correctly rejected non-object instances, FP (false positive) refers to incorrectly detected objects (false alarms), and FN (false negative) is the number of missed detections.

In addition to these basic metrics, we incorporated sensitivity and specificity to further evaluate the model’s robustness across different classes. Sensitivity is identical to recall and reflects the model’s ability to identify all relevant objects, whereas specificity (true negative rate) measures the reliability of correctly identifying non-object instances or background noise.

These metrics are formulated as follows:

(49) $$Sensitivity=\frac{TP}{TP+FN}\;,$$

(50) $$Specificity=\frac{TN}{TN+FP}\;.$$

Furthermore, the mean average precision (mAP50) is employed to evaluate the accuracy of both classification and localisation. It is calculated as the mean of the average precision (AP) across all classes, where a detection is considered a true positive if the intersection over union (IoU) between the predicted and ground truth bounding box is at least 0.5.

The mAP is defined as

(51) $$mAP=\frac{1}{n}\sum_{i=1}^{n}AP_{i}\;.$$

Finally, to evaluate the model’s performance across all possible classification thresholds, we utilized the area under the receiver operating characteristic curve (AUROC) and the area under the precision–recall curve (AUPRC). The AUROC illustrates the trade-off between the true positive rate (TPR) and the false positive rate (FPR), providing a measure of the model’s ability to distinguish between classes. The AUPRC is particularly useful for highly imbalanced datasets, as it focuses on the performance of the positive class by calculating the area under the precision-versus-recall curve as

(52) $$AUROC=\int_{0}^{1}TPR\left(FPR\right)d\left(FPR\right)\;,$$

(53) $$AUPRC=\int_{0}^{1}Precision\left(Recall\right)d\left(Recall\right)\;.$$

These comprehensive metrics are used to compare the proposed method with existing frameworks in order to quantitatively evaluate the performance improvements achieved in various detection environments.

## 4. Experimental Results and Performance

**Figure 16:**
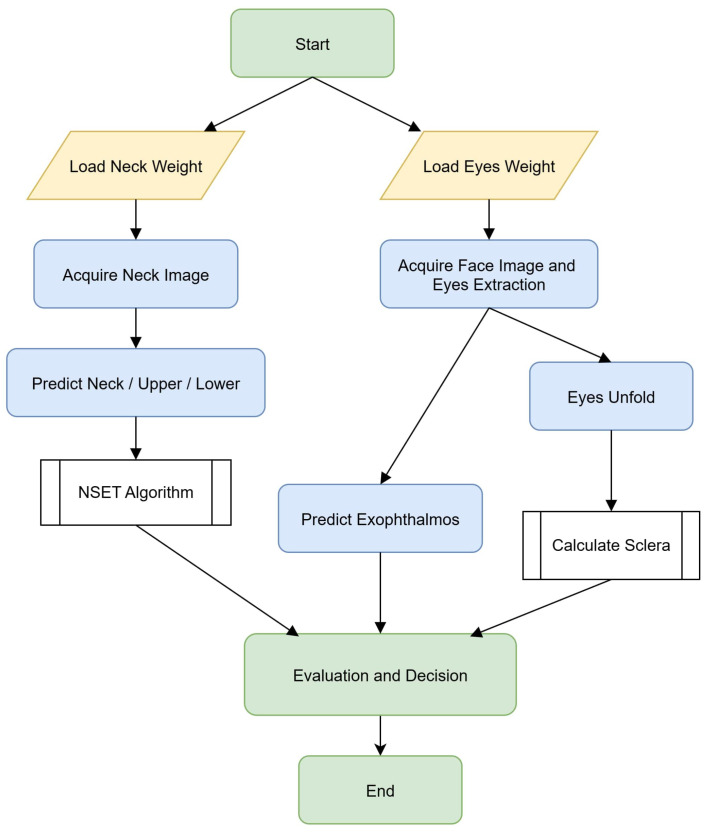
Workflow of the proposed system integrating neck and eye modules for clinical evaluation.

As demonstrated in Figure 16, the proposed system initiates the diagnostic process by loading the pretrained weights for both the neck and eye image datasets. The workflow proceeds as follows. For the neck module, the system acquires the neck image and performs classification into the neck, upper neck, or lower neck categories, with the results undergoing further analysis using the NSET algorithm. For the eye module, facial images are processed via the eye extraction method followed by the eye unfold procedure to normalize scleral features and calculate relevant parameters. This module also concurrently performs the prediction of exophthalmos.

The outcomes from both specialized modules were then combined in the evaluation and decision stage to generate a comprehensive and integrated analysis. This unified workflow highlights the seamless integration of the neck and eye modules within a single system, thereby significantly enhancing the accuracy and reliability of clinical image-based evaluation for thyroid-associated disorders.

The experimental parameter configurations, encompassing data preprocessing methods, data augmentation techniques, and hyperparameter settings for model training, are summarized in Table 1. All parameters were standardized across all models to ensure consistency and facilitate accurate reproducibility of the experiments. The evaluated architectures were the baseline CNN, ResNet18, EfficientNet-B0, MobileNetV2, MobileNetV3 Small, and DenseNet121. The ImageNet-pretrained models were trained with the backbone frozen, and only the classification head was fine-tuned according to finetune_head_only, while the baseline CNN was trained end-to-end from scratch.

### 4.1. Sclera Calculation Results

**Figure 17:**
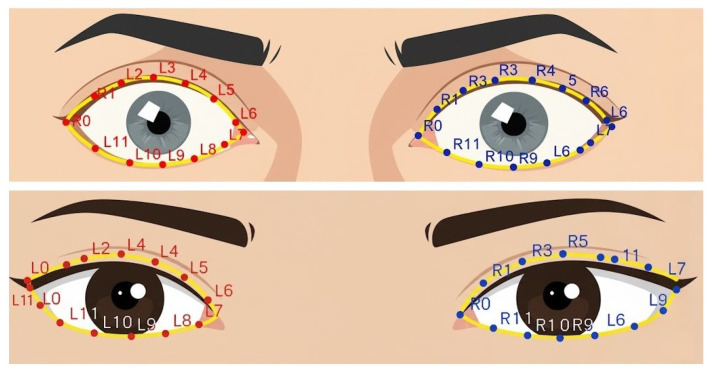
Landmark results: (**upper**) thyroid-associated and (**lower**) normal eye.

Figure 17 demonstrates the assignment of reference landmarks around the eyes for scleral size measurement. The left eye was defined with landmarks L0–L11, and the right eye was defined with R0–R11, all of which were derived from geometric image processing calculations. These landmarks served as guiding points for drawing straight lines inward toward the iris, enabling precise measurement of the distance from the eyelid boundary to the iris margin at each designated position. This approach provides a standardized and detailed quantification of the scleral size. The comparative evaluation between patients with early signs of exophthalmos and normal eyes highlights significant morphological differences that carry high clinical relevance.

**Figure 18:**
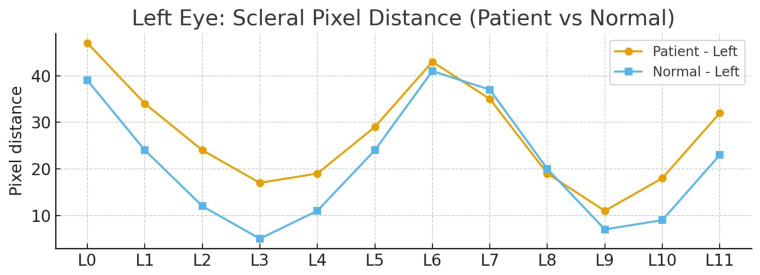
Comparison of left eye scleral pixel distances between patient and normal groups.

Table 2 summarizes the pixel distance measurements for both the left (L0–L11) and right (R0–R11) eyes, comparing two distinct groups. As demonstrated in Figure 18, the comparison for the left eye revealed that the first group consistently exhibited higher values across almost all designated positions compared with the normal group. The left eye exhibited an average difference of 6.33 pixels and a total difference of 76 pixels compared with the normal group. The most prominent disparity occurred at position L2 with a 12-pixel difference, indicating a pronounced scleral expansion in the patient’s left eye, which is highly consistent with the manifestation of thyroid-associated exophthalmos.

**Figure 19:**
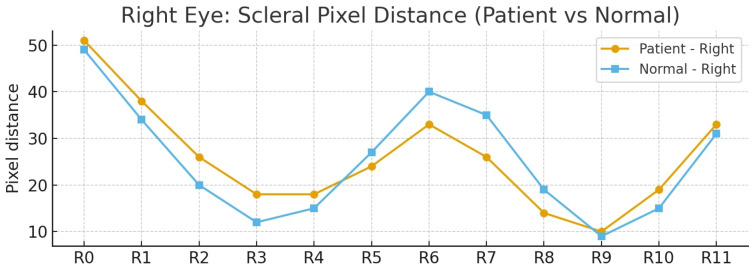
Comparison of right eye scleral pixel distances between patient and normal groups.

Figure 19 presents the right eye comparison. The overall average values between the patients and normal individuals were highly similar, showing a total difference of only 4 pixels and an average difference of 0.33 pixels. Nevertheless, localized variations were evident. Certain positions showed the patient’s sclera to be larger, such as R2 with a six-pixel difference, and others where the difference was minimal, reflecting greater balance in the right eye overall. This final observation highlights the significant asymmetry between the two eyes, with a left eye average difference of 6.33 pixels versus a right eye average difference of 0.33 pixels, which is a key clinical characteristic often seen in the early stages of thyroid-associated ophthalmopathy.

**Figure 20:**
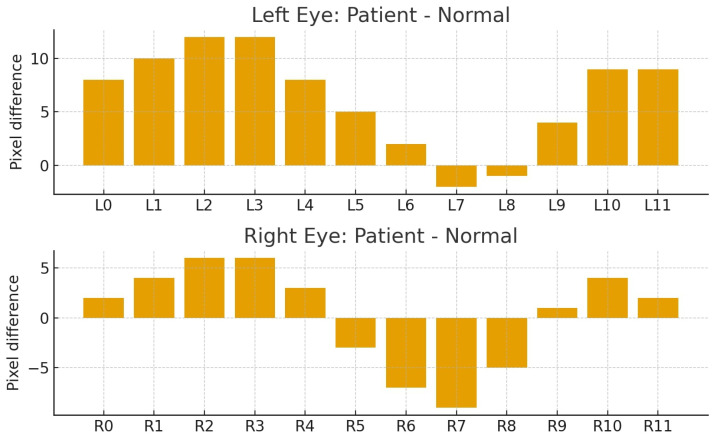
Bar plot of pixel distance differences between patient and normal groups for (**upper**) left and (**lower**) right eyes.

Figure 20 presents a bar plot summarizing the pixel differences in scleral measurements between the patient and normal eyes for both the left and right sides. The left eye {L0–L11} showed predominantly positive values, indicating consistent expansion of the scleral region compared with the normal eye across most positions. The right eye {R0–R11} exhibited alternating positive and negative differences, reflecting a more balanced, localized pattern of change. This pattern emphasizes that the patient’s ocular alterations were more consistently and strongly evident in the left eye, thereby making it a more robust clinical indicator of pathology in this specific case.

In summary, the selection of positions {L0–L11} and {R0–R11} as reference points for scleral measurement allowed for a detailed spatial analysis of the ocular morphology. The combined results across Figure 18 and Figure 19 consistently highlight that the patients’ left eyes showed more consistent and pronounced changes. This observed inter-eye asymmetry and the consistent morphological difference serve as a critical criteria for distinguishing patients with early thyroid-associated ophthalmopathy from normal individuals.

### 4.2. Evaluating Integrated CNN-SMUE Features for Eye Dataset Prediction

The dataset utilized in this study consisted of 230 eye image samples systematically categorized into two groups: 131 normal images and 99 patient images. The normal class samples were sourced from the publicly available CelebFaces Attributes dataset [30], while the patient class images were obtained from a private dataset collected from individuals undergoing medical evaluation.

The composition of the dataset is detailed in Table 3, showcasing a diverse distribution across sex and age ranges. Specifically, the data included 120 males and 110 females, with ages spanning from under 9 to over 60 years. The most significant concentration of data was within the 20–29-year age range, comprising 72 samples. To ensure clinical reliability, the labeling process was conducted under the supervision of medical experts. Each image was carefully annotated with precise bounding box coordinates and class labels to facilitate effective feature extraction of morphological eye characteristics. Although the normal class slightly outnumbered the patient class, the overall distribution was maintained to mitigate the risk of class imbalance and prevent model bias toward the majority class. This structured data design enabled the deep learning models to accurately distinguish between healthy individuals and patients with specific conditions.

All images were meticulously categorized into two distinct groups: normal and hyperthyroid. The crucial labeling process was performed under the direct supervision of medical experts to ensure robust clinical accuracy. The complete dataset was systematically partitioned into dedicated training and test sets for sequential model development and rigorous performance evaluation. Each image was stored alongside a corresponding label file adhering to the YOLO dataset structure, which includes precise bounding box coordinates and object class annotations. This specific dataset design enabled the deep learning model to effectively capture fine morphological eye features and clearly distinguish between normal individuals and patients with thyroid-related conditions.

**Figure 21:**
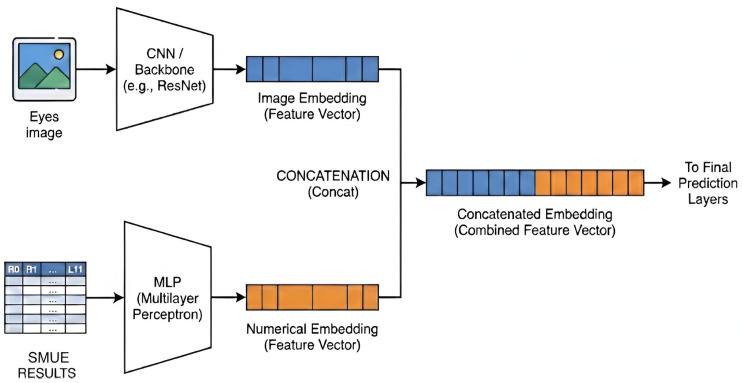
The dual-branch architectural framework for feature extraction and concatenation.

Before commencing the model training process, it was essential to integrate the numerical features derived from the SMUE results with the visual eye images. This data fusion step ensured that the model could learn from a comprehensive feature set during the training phase. The architectural process for this integration is illustrated in Figure 21. This figure demonstrates the hybrid model architecture employed during the experimental phase to integrate multimodal data. The image branch utilizes a CNN backbone to extract high-dimensional embeddings from eye images. Simultaneously, the SMUE results branch processes numerical features (ranging from R0 to L11) through a multilayer perceptron (MLP). The latent representations from both branches are subsequently concatenated to form a unified feature vector, which serves as the comprehensive input for the final prediction layers.

This integration allows the model to leverage both spatial visual information and quantitative SMUE data to achieve the reported performance. The core objective of this experimental phase was to evaluate the effectiveness of integrating multi-modal data to enhance classification accuracy. By comparing standard deep learning architectures with their augmented counterparts, this study aims to demonstrate the quantitative impact of incorporating SMUE numerical features into the visual feature extraction process. Before the training phase, data from SMUE results were systematically integrated with the eye images through the dual-branch architecture previously described. The following section details the comparative performance of these models across multiple evaluation metrics, highlighting the significant disparities between standalone image-based processing and the proposed fusion-based approach. The comprehensive results are summarized in Table 4.

Table 4 provides an extensive evaluation of various deep learning architectures, comparing their performance as standalone image classifiers against their performance when integrated with numerical SMUE data. The evaluation was expanded to include eight key performance metrics: precision, recall, accuracy, mAP50, AUROC, AUPRC, sensitivity, and specificity. The results indicate that the standalone models relying solely on eye images exhibited moderate performance.

For instance, in Table 4, EfficientNet-B0 recorded the lowest accuracy at 71.4%, while YOLOv11s demonstrated the strongest standalone performance with an accuracy of 89.1% and a high sensitivity of 95.2%.

A significant performance breakthrough was observed upon the integration of SMUE numerical features through the proposed dual-branch architecture. By concatenating image embeddings with numerical features, the predictive power across nearly all architectures improved substantially. Notably, the CNN + SMUE, MobileNetV2 + SMUE, and MobileNetV3 + SMUE configurations achieved near-optimal results, reaching 98.9% across all evaluation metrics, including the AUROC and specificity. Regarding the YOLOv11s + SMUE configuration, it remained excluded from the multimodal integration.

Due to YOLO’s fundamental end-to-end object detection architecture, it does not inherently support the late-stage feature fusion or vector concatenation employed in this study. Consequently, while YOLOv11s is a robust standalone model, it is technically incompatible with the proposed framework, which requires flexible latent feature extraction for multimodal fusion.

**Figure 22:**
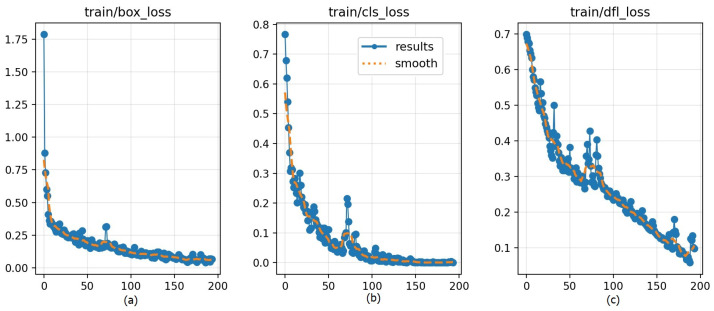
Training and validation box loss across epochs of eyes dataset: (**a**) box regression loss (train/box_loss); (**b**) classification loss (train/cls_loss) and (**c**) distribution focal loss (train/dfl_loss).

Based on the comparative results in the table, the CNN + SMUE configuration was selected as the representative model due to its consistently high performance across all reported metrics. Figure 22 summarizes the training convergence over 200 epochs using three loss components. Figure 22a train/box_loss shows a sharp decrease during the early epochs before gradually stabilizing at a low value, indicating improved localisation accuracy and convergence of the regression term. Figure 22b train/cls_loss also dropped rapidly and approached near-zero values in later epochs, suggesting that class discrimination became stable after sufficient training. Finally, Figure 22c train/dfl_loss exhibits a steady downward trend with moderate fluctuations, reflecting progressive refinement of box quality estimation.

**Figure 23:**
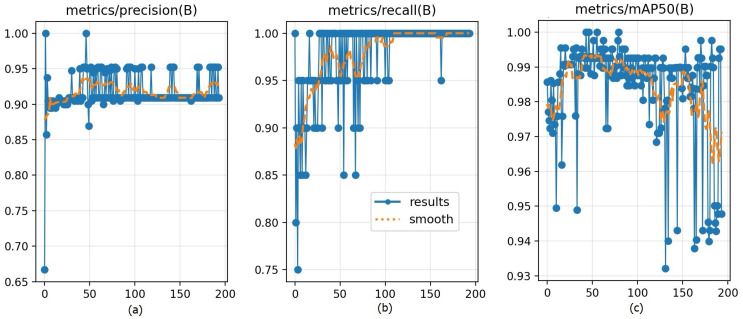
Precision, recall, and mAP50 performance curves across training epochs for the eye dataset: (**a**) precision, (**b**) recall, and (**c**) mAP50.

Figure 23 reports the validation performance across epochs. Figure 23a metrics/precision(B) increased from an initially unstable regime to a stable plateau around a high precision level, implying reduced false positives as training proceeded. Figure 23b metrics/recall(B) rose rapidly and approached saturation near 1.0, indicating that most ground-truth instances were successfully retrieved after the mid-training stage. Lastly, Figure 23c metrics/mAP50(B) remained high at approximately 0.98 for the majority of epochs, demonstrating strong overall detection quality at IoU = 0.50.

Despite the technical incompatibility of YOLOv11s with SMUE features, this study utilized YOLOv11s to identify and localize ROIs within the experimental images, thereby effectively defining the scope for data analysis. Based on the experimental results, YOLOv11s demonstrated the highest performance among all standalone models. Furthermore, its selection was justified by its continuous development and state-of-the-art status in current research.

### 4.3. NSET Algorithm Results for Neck Dataset

The dataset utilized in this study consisted of 230 image samples systematically categorized into two groups: 130 normal images and 100 patient images. The samples in the normal class were sourced from the CelebFaces Attributes dataset [30], while the patient class images were obtained from a private dataset collected from individuals exhibiting clinical symptoms. The composition of the dataset is detailed in Table 5, showcasing a diverse distribution across sex and age ranges to ensure demographic representativeness. Specifically, the data included 73 male and 157 female subjects, with ages spanning from under 9 to over 60 years. The most significant concentration of data was observed within the 20–29-year age range, which comprised 72 samples, followed by the 30–39-year age range with 46 samples. This structured distribution across various age cohorts and genders allowed the model to minimize potential confounding variables and enhanced the robustness of the diagnostic framework.

All images were annotated using bounding boxes to define the regions of interest and categorized into two classes: TopNeck, representing the upper portion of the neck, and BelowNeck, representing the lower portion of the neck. The dataset was organized in YOLO-compatible format, where each image was accompanied by a text annotation file containing bounding box coordinates and class identifiers. This dataset structure was designed to enable the model to effectively learn the morphological differences between various sub regions of the neck. Furthermore, the dataset was divided into training and testing subsets, ensuring that the trained model could achieve high accuracy while maintaining unbiased performance evaluation.

Based on the empirical evidence presented in Table 6, YOLOv11s was selected as the primary model for identifying specific anatomical regions, including BelowNeck and TopNeck. The decision was justified by its superior performance across all evaluated metrics compared with traditional classification architectures.

While standard models such as the CNN, ResNet18, and MobileNet variants struggled with this specific task, recording accuracy scores as low as 46.7% and 73.3%, YOLOv11s achieved a significantly higher accuracy of 89.9%. Furthermore, its mAP50 of 95.7% and recall of 92.5% demonstrate a robust capability in localizing and distinguishing complex features within the neck images. The end-to-end nature of the YOLO framework provides a distinct advantage in capturing spatial context, making it the most reliable candidate for precise regional identification in this research.

Following the precise localisation of anatomical regions using YOLOv11s, as presented in Table 6, the extracted coordinates for the TopNeck and BelowNeck segments underwent a specialized statistical evaluation. This next phase employs the proposed NSET method. By calculating the statistical variance between the TopNeck and BelowNeck regions, the proposed NSET algorithm can objectively distinguish pathological swelling from normal physiological variations. The comparative performance of this statistical ensemble approach against various deep learning baselines is demonstrated in Table 6, where NSET exhibited superior accuracy in identifying swollen conditions.

Table 7 presents a comprehensive performance analysis for the classification of “swollen” and “normal” neck conditions, comparing the proposed NSET against various deep learning baselines. The experimental results reveal that NSET stood as the most effective methodology, achieving the highest scores across all metrics, with a precision of 88.4%, recall of 87.5%, accuracy of 87.5%, and mAP50 of 92.0%. This superior performance significantly outpaced all neural network architectures evaluated in this study. Among the high-performance baselines, YOLOv11s achieved an accuracy of 81.9% and mAP50 of 83.5%, while EfficientNet-B0 followed with an accuracy of 71.4%.

In the mid-range tier shown in Table 7, both MobileNetV2 and MobileNetV3 demonstrated identical accuracy results at 64.3%, though MobileNetV2 struggled, with a lower mAP50 of 51.8% compared with MobileNetV3’s 58.1%. Conversely, standard models such as the CNN and ResNet18 exhibited lower reliability, with an accuracy of only 57.1%.

The least effective performance was observed for DenseNet121, which yielded an accuracy of 35.7% and precision of only 17.9%. A critical factor contributing to the success of NSET is its nature as a statistical ensemble technique that does not require a data training phase. Unlike the deep learning models, which demand extensive training and large datasets to optimize parameters, NSET effectively bypasses these requirements, making it exceptionally well suited for specialized clinical datasets with limited samples, where traditional deep learning models often fail to generalize or suffer from overfitting.

## 5. Discussion

**Figure 24:**
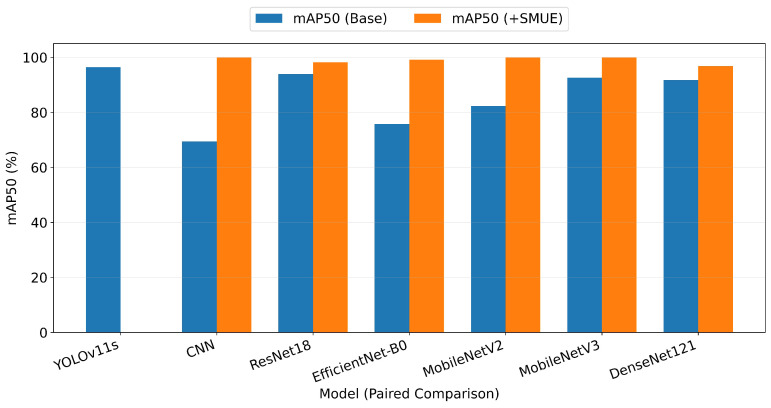
Comparative analysis of mAP50 between baseline deep learning models and those integrated with SMUE process.

The experimental results demonstrate that integration of the SMUE process significantly enhanced the diagnostic performance across all evaluated deep learning models. Figure 24 presents a comparative analysis of the mAP50 scores between the baseline models and those integrated with the proposed SMUE framework. The empirical results demonstrate a significant performance elevation across most architectures. Notably, the CNN model exhibited a substantial increase of 30.5%, rising from a baseline of 69.4% to 99.9% post integration.

Similarly, MobileNetV2 showed an improvement of 17.6%, achieving a near-perfect mAP50 of 99.9%. It should be noted that while YOLOv11s demonstrated strong baseline performance, its evaluation with SMUE was precluded due to specific operational constraints, and thus only its baseline data were reported. Nevertheless, the quantitative metrics for other SMUE-augmented models, particularly within the MobileNet family, consistently reached 99.9% for precision, recall, and accuracy.

These results significantly outperformed standard high-capacity architectures such as ResNet18 and DenseNet121 in their respective baseline states. In conclusion, the integration of SMUE yielded an average performance boost from 10% to 30%. This validates the framework’s efficacy in elevating lightweight architectures to high-precision medical diagnostic standards without necessitating excessive computational complexity.

**Figure 25:**
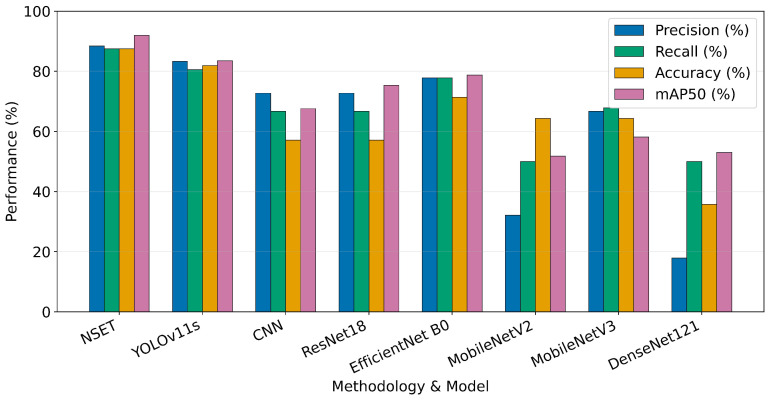
Comparative diagnostic performance of the NSET algorithm versus various deep learning models for neck image classification.

Figure 25 presents a comparative evaluation of the diagnostic performance across all tested methodologies, highlighting that the proposed NSET algorithm provided the most optimal results for neck image classification. NSET consistently outperformed other methods across all metrics, with its precision, recall, and accuracy ranging between 87.5 and 88.4% and achieving an mAP50 of 92.0%.

Within the deep learning baselines, YOLOv11s delivered the best results, with an mAP50 of 83.5%. However, its performance remained markedly lower than NSET under the same evaluation conditions. Conversely, more complex architectures such as DenseNet121 exhibited significant instability and performance degradation in this task, recording the lowest precision at only 17.9%. In conclusion, these experimental results support that NSET not only enhances diagnostic accuracy but is also highly practical for small-scale clinical datasets, offering reliable classification without the intensive computational and training requirements typical of deep learning models.

## 6. Conclusions

This research successfully presented a comprehensive framework for personal image analysis by integrating a tripartite evaluation approach focusing on the eye and neck regions. The system effectively combines eye image prediction using the preprocessing YOLOv11s deep learning model with eye area analysis through the sclera mapping and unwrapping extraction (SMUE) algorithm and neck swelling evaluation via the NSET algorithm, the latter of which demonstrated a superior mAP50 of 92.0% on limited datasets. By aggregating these three indicators and transmitting the results to experts for final consideration, the framework not only enhances analysis precision and reliability but also offers a resource-efficient solution that bypasses intensive training requirements. Ultimately, this methodology serves as a robust and practical decision support tool, being particularly effective in environments with data and computational constraints.

However, several limitations within the present study remain to be addressed. Firstly, the image acquisition process requires participants to maintain a direct frontal orientation toward the camera. While preprocessing techniques are employed to standardize image dimensions, inconsistent capture distances can lead to suboptimal clarity, potentially compromising the accuracy of the SMUE algorithm. Furthermore, ambient lighting control is a critical factor; both eye and neck analysis rely heavily on precise illumination to accurately evaluate scleral area and cervical swelling.

Consequently, lighting variations represent a significant challenge that impacts experimental outcomes. These constraints provide a clear roadmap for enhancing the robustness of future research. Additionally, while YOLO remains a highly promising framework due to its superior predictive performance and rapid processing speed, it currently lacks a standardized mechanism for direct integration into combined feature vector methods within this study’s context. Developing a seamless integration between YOLO-based localisation and feature vector fusion thus represents another compelling direction for future investigation.

## Figures and Tables

**Table 1:** Model training and preprocessing parameters.

Category	Parameter or Method	Value or Detail
Image preprocessing	Input resolution	320×320 pixels
	and normalization	ImageNet statistics:
		μ={0.485,0.456,0.406}
		σ={0.229,0.224,0.225}
Data augmentation	Techniques	Random horizontal flip
Optimization	Optimizer	AdamW
	Learning rate	$1\times 10^{-3}$
	Weight decay	$1\times 10^{-4}$
Training settings	Batch size	16
	Maximum epochs	1000
	Early stopping	Patience of 100 epochs
	Precision control	Automatic mixed precision (AMP) enabled
Class imbalance	Handling method	WeightedRandomSampler
	Loss function	Class-weighted cross-entropy loss

**Table 2:** Scleral distance measurements at each position for patient eyes and normal eyes.

Class	Left Eye Pixel Distance	Right Eye Pixel Distance
Patient	L0 = 47, L1 = 34, L2 = 24, L3 = 17, L4 = 19, L5 = 29, L6 = 43, L7 = 35, L8 = 19, L9 = 11, L10 = 18, L11 = 32	R0 = 51, R1 = 38, R2 = 26, R3 = 18, R4 = 18, R5 = 24, R6 = 33, R7 = 26, R8 = 14, R9 = 10, R10 = 19, R11 = 33
Normal	L0 = 39, L1 = 24, L2 = 12, L3 = 5.0, L4 = 11, L5 = 24, L6 = 41, L7 = 37, L8 = 20, L9 = 7.0, L10 = 9, L11 = 23	R0 = 49, R1 = 34, R2 = 20, R3 = 12, R4 = 15, R5 = 27, R6 = 40, R7 = 35, R8 = 19, R9 = 9, R10 = 15, R11 = 31

**Table 3:** Eye dataset composition by class, sex, and age.

Characteristic	Total	Patient	Normal
Sex:			
Male	120	49	71
Female	110	50	60
Age range (years):			
0–9	8	4	4
10–19	17	6	11
20–29	72	30	42
30–39	48	21	27
40–49	40	13	27
50–59	25	14	11
60+	20	11	9
Total	230	99	131

**Table 4:** Performance comparison of deep learning models with and without SMUE integraton for eye image classification.

Model	Model	Precision	Recall	Accuracy	mAP50	AUROC	AUPRC	Sensitivity	Specificity
Type	Name	(%)	(%)	(%)	(%)	(%)	(%)	(%)	(%)
Baseline	CNN	73.6	73.3	73.8	69.4	73.3	74.1	78.5	72.1
models	ResNet18	85.6	85.6	85.7	93.9	85.6	85.8	91.1	84.5
	EfficientNetB0	74.8	73.0	71.4	75.7	73.1	70.1	76.5	74.2
	MobileNetV2	76.4	75.2	73.8	82.3	75.2	72.5	79.1	75.5
	MobileNetV3	85.6	85.6	85.7	92.6	85.6	85.8	90.5	84.5
	DenseNet121	84.7	84.3	83.3	91.7	84.3	82.5	88.3	83.5
	YOLOv11s	91.1	94.6	89.1	96.4	94.6	83.5	95.2	90.1
Proposed	CNN+SMUE	98.9	98.9	98.9	98.9	98.9	98.9	98.9	98.9
method	ResNet18	84.9	84.9	85.1	91.5	91.5	90.8	85.7	84.2
	+ SMUE								
	EfficientNet	87.5	87.6	87.5	98.4	98.9	98.9	98.9	98.9
	B0 + SMUE								
	MobileNetV2	98.9	98.9	98.9	98.9	98.9	98.9	98.9	98.9
	+ SMUE								
	MobileNetV3	98.9	98.9	98.9	98.9	98.9	98.9	98.9	98.9
	+ SMUE								
	DenseNet121	93.2	92.9	92.5	95.1	96.7	98.1	90.5	98.9
	+ SMUE								

**Table 5:** Neck dataset composition by class, sex, and age.

Characteristic	Total	Patient	Normal
Sex:			
Male	73	32	41
Female	157	68	89
Age range (years):			
0–9	16	1	15
10–19	33	16	17
20–29	72	34	38
30–39	35	13	22
40–49	31	15	16
50–59	21	10	11
60+	22	11	11
Total	230	100	130

**Table 6:** Performance benchmark of deep learning algorithms on neck image classification as {BelowNeck, TopNeck}.

Model	Precision (%)	Recall (%)	Accuracy (%)	mAP50 (%)
YOLOv11s	87.6	92.5	89.9	95.7
CNN	31.0	46.7	46.7	62.5
ResNet18	65.6	66.7	66.7	75.3
EfficientNet B0	68.3	66.7	66.7	76.0
MobileNetV2	73.8	73.3	73.3	86.1
MobileNetV3	72.8	73.3	73.3	80.5
DenseNet121	81.0	73.3	73.3	72.3

**Table 7:** Comparative performance of baseline deep learning models for neck image classification as {Swollen, Normal}.

Model	Precision (%)	Recall (%)	Accuracy (%)	mAP50 (%)
NSET	88.4	87.5	87.5	92.0
YOLOv11s	83.3	80.6	81.9	83.5
CNN	72.7	66.7	57.1	67.6
ResNet18	72.7	66.7	57.1	75.3
EfficientNet B0	77.8	77.8	71.4	78.7
MobileNetV2	32.1	50.0	64.3	51.8
MobileNetV3	66.7	67.8	64.3	58.1
DenseNet121	17.9	50.0	35.7	53.0

## Data Availability

The data presented in this study are available in [30].

## Abbreviations

The following abbreviations are used in this manuscript:

AI	Artificial intelligence
AP	Average precision
AUPRC	Area under the precision–recall curve
AUROC	Area under the receiver operating characteristic curve
ASR	Asymmetry ratio
BAR	Bulge area ratio
BPI	Bulge peak index
CLAHE	Contrast limited adaptive histogram equalization
CNN	Convolutional neural network
FMEL	Face mesh-based eye landmark
FN	False negative
FP	False positive
FPR	False positive rate
IoU	Intersection over union
mAP	Mean average precision
MLP	Multilayer perceptron
NSET	μ−σ ensemble thresholding
PF	Palpebral fissure
ROI	Region of interest
SMUE	Sclera map unwrapping engine
TED	Thyroid eye disease
TLR	Top-to-low width ratio
TN	True negative
TP	True positive
TPR	True positive rate
